# Calcimimetic restores diabetic peripheral neuropathy by ameliorating apoptosis and improving autophagy

**DOI:** 10.1038/s41419-018-1192-7

**Published:** 2018-11-26

**Authors:** You Chul Chung, Ji Hee Lim, Hyun Mi Oh, Hyung Wook Kim, Min Young Kim, Eun Nim Kim, Yaeni Kim, Yoon Sik Chang, Hye Won Kim, Cheol Whee Park

**Affiliations:** 1 Department of Rehabilitation Medicine, College of Medicine, The Catholic University of Korea, Seoul, Korea; 2 Division of Nephrology, Department of Internal Medicine, College of Medicine, The Catholic University of Korea, Seoul, Korea; 3 Institute for Aging and Metabolic Diseases, College of Medicine, The Catholic University of Korea, Seoul, Korea

## Abstract

Decreased AMPK-eNOS bioavailability mediates the development of diabetic peripheral neuropathy (DPN) through increased apoptosis and decreased autophagy activity in relation to oxidative stress. Schwann cells are responsible for maintaining structural and functional integrity of neurons and for repairing damaged nerves. We evaluated the neuro-protective effect of cinacalcet on DPN by activating the AMPK-eNOS pathway using *db/db* mice and human Schwann cells (HSCs). Sciatic nerve of *db/db* mice was characterized by disorganized myelin, axonal shrinkage, and degeneration that were accompanied by marked fibrosis, inflammation, and apoptosis. These phenotypical alterations were significantly improved by cinacalcet treatment along with improvement in sensorimotor functional parameters. Cinacalcet demonstrated favorable effects through increased expression and activation of calcium-sensing receptor (CaSR)-CaMKKβ and phosphorylation of AMPK-eNOS signaling in diabetic sciatic nerve. Cinacalcet decreased apoptosis and increased autophagy activity in relation to decreased oxidative stress in HSCs cultured in high-glucose medium as well. This was accompanied by increased expression of the CaSR, intracellular Ca^++^ ([Ca^++^]i) levels, and CaMKKβ-LKB1-AMPK signaling pathway, resulting in the net effect of increased eNOS phosphorylation, NOx concentration, Bcl-2/Bax ratio, beclin 1, and LC3-II/LC3-I ratio. These results demonstrated that cinacalcet treatment ameliorates inflammation, apoptosis, and autophagy through increased expression of the CaSR, [Ca^++^]i levels and subsequent activation of CaMKKβ-LKB-1-AMPK-eNOS pathway in the sciatic nerve and HSCs under diabetic condition. Therefore, cinacalcet may play an important role in the restoration and amelioration of DPN by ameliorating apoptosis and improving autophagy.

## Introduction

Diabetic peripheral neuropathy (DPN) is one of the most common complications of diabetes in >50–60% of all diabetic patients, and it is also the leading cause of amputation worldwide^1,2^. The early changes in patients with DPN include accumulation of extracellular matrix proteins, inflammation, axonal degeneration, and loss of unmylelinated fibers, which cause sensorimotor conduction delays and irreversible nerve damage. It is well known that hyperglycemia plays a main role in DPN^3–7^ with regard to the changes in oxidative–nitrosative stress, neuro-inflammation, mitochondrial dysfunction, bio-energetic crisis, and demyelination^7^.

Schwann cells (SCs) are specialized glial cells in the peripheral nervous system that are responsible for maintaining structural and functional integrity of neurons and for repairing damaged nerves^8,9^. Hyperglycemia-induced SC damages may reduce nerve conduction velocity, accelerate axonal atrophy, and impair axonal regeneration^10^. Moreover, hyperglycemia-induced SC damages include such morphological changes as swelling and vacuolization that result in the destruction of organelles. Clearance of defective organelles constitutes the very core of the autophagy process that is an important physiological and defensive mechanism of the cell and body under such deranged metabolic conditions as nutrient or energy excess and deprivation^11^. Chronic hyperglycemia with diabetes impairs cellular autophagy and exacerbates apoptosis associated with DPN^7,12^. Autophagy promotes cell survival by sequestering senescent or damaged organelles/proteins in autophagosomes for recycling of their products^11^. Therefore, an enhancement of autophagy and a concomitant suppression of apoptosis of SCs might be the optimal strategy for the prevention and regression of DPN.

AMP-activated protein kinase (AMPK) is a master controller of cellular energy balance that activates catabolic pathways in state of energy deprivation^13^. Chronic nutrient excess state associated with prolonged diabetes triggers a switching off of AMPK, which results in impaired peroxisome proliferator-activated receptor γ coactivator-1α (PGC-1α) activity and diminished mitochondrial^14^ and endothelial nitric oxide synthase (eNOS) activities^15^, leading to neurodegeneration in patients with DPN. The important mode of AMPK activation relies on phosphorylation at the 172nd threonine residue of the α-subunit by upstream kinases, including Ca^++^/calmodulin-dependent protein kinase kinase β (CaMKKβ) and liver kinase B1 (LKB1). The LKB1 forms a complex with STRAD and MO25 in response to an elevation in AMP/ATP ratio^16^, which phosphorylates the AMPKα subunit to trigger the AMPK pathway. CaMKKβ is an alternative upstream kinase of AMPK that responds to the change in intracellular Ca^++^ ([Ca^++^]i) concentration. Elevated [Ca^++^]i increases the activity of AMPK, independent of the adenylate energy balance^17^.

The calcimimetic, (R)-N-(3-(3-(trifluoromethyl)phenyl)propyl)-1-(1-napthyl)ethylamine hydrochloride (cinacalcet), devised originally for the treatment of secondary hyperparathyroidism, exerts its effect by stimulating Ca^++^-sensing receptor (CaSR) mainly in the parathyroid glands^18^. Activated upon Ca^++^ ions, the expression of cellular surface CaSR is crucial for maintaining a stable serum Ca^++^, which is achieved primarily through the regulation of parathyroid hormone secretion and renal Ca^++^ excretion. Interestingly, the expression of CaSR has been demonstrated in the vasculature and perivascular sensory nerves^18,19^. CaSR activation by cinacalcet is known to activate CaMMK-LKB1-AMPK pathway. Activation of AMPK and LKB-1 is crucial for SC-mediated axonal maintenance while LKB deletion is responsible for axonal degeneration^20^. Moreover, the CaSR is known to modulate cell proliferation and apoptosis and coordinate oncogene expression, chemotaxis, and autophagy. Exposed under constant metabolic stress, these axons and SCs are prone to mitochondrial dysfunction featuring derangements in [Ca^++^]i homeostasis and associated downstream signaling that are key causal factors for the development of DPN, making it an ideal therapeutic target at the same time^21^.

To date, there is no curative therapy currently available to deter the progression of DPN; only a handful of studies have demonstrated improvements in indices of neuropathy through the activation of AMPK in cultured neurons^22^ and peripheral nerves of type 1 diabetic rats^23^. On this account, we assumed that cinacalcet treatment may modulate DPN activity through the axis delineated in the previous study. Thus we investigated the protective and/or reversal effect of cinacalcet against neural glucotoxicity through the changes in the AMPK-eNOS pathway in the sciatic nerve of *db/db* mice and human Schwann cells (HSCs).

## Materials and methods

### Experimental animals and assessment of peripheral nerve function

All animal experiments were performed in accordance with the Laboratory Animals Welfare Act, the *Guide for the Care and Use of Laboratory Animals*, and were approved by the Institutional Animal Care and Use Committee (IACUC) at College of Medicine, the Catholic University of Korea (CUMC-2014^−^0165-01). Eight-week-old male C57BLKS/J *db/m* and *db/db* mice were purchased from Jackson Laboratories (Bar Harbor, ME, USA) and *db/m* and *db/db* mice were divided into four groups. Cinacalcet (10 mg/kg) mixed into standard chow diet or a regular diet was administered to *db/db* mice (*db/db*+cina; *n* = 8) and age- and gender-matched *db/m* mice (*db/*m+cina; n = 8) for 12 weeks starting at 8 weeks of age.

After 12 weeks of cinacalcet treatment, we performed electrophysiological and sensory threshold tests in the following order: tactile responses (a response 50% of the times the tip is applied to the hind paw) to stimulation using flexible von Frey filaments and then sciatic motor nerve conduction latency (MNCL), as described previously^15^. After the tests were completed, blood was collected from the left ventricle and the plasma was stored at −70 °C for subsequent analyses, and we collected the sciatic nerves under general anesthesia with 10 mg/kg xylazine hydrochloride (Rompun; Bayer, Leuverkusen, Germany) and 30 mg/kg tiletamine plus zolazepam (Zoletil; Virbac, Carros, France). Some sciatic nerve samples were fixed in normal buffered 4% formalin for immunohistochemistry, and the others were stored in a solution for electron microscopy. We also collected the sciatic nerves in 8-week old male *db/m* and *db/db* mice for evaluation of cinacalcet effect on the recovery of DPN.

### Assessment of blood glucose, HbA1c, plasma ionized calcium, and PO_4_^−^ concentrations

After 12 weeks of treatment with cinacalcet, blood glucose was measured using an Accucheck meter (Roche Diagnostics, St. Louis, MO). Hemoglobin A1c (HbA1c) was determined on red cell lysates using high-performance liquid chromatography (BioRad, Hercules, CA). Plasma ionized calcium (iCa^++^) and PO_4_^−^ concentrations were measured using colorimetry (Samkwang Medical Laboratory, Seoul, Korea).

### Light and electron microscopic analysis

#### Nerve morphology

Sciatic nerve samples were fixed in 4% paraformaldehyde. Trichrome-stained nerves were used to examine the effect of cinacalcet on nerve fibrosis. Ten consecutive nerve cross-sections were photographed using a digital camera (Olympus DP11; Olympus America, Melville, NY) by an examiner who was blinded to the tissue source. Each nerve section was sampled in a serpentine pattern such that the entire nerve section was analyzed with no overlapping fields. We performed immunohistochemistry for type IV collagen (Col IV; Biodesign International, Saco, ME, USA) and 8-hydroxy-deoxyguanosine (8-OH-dG; JalCA, Fukuroi, Shizuoka, Japan), an oxidative DNA damage marker.

#### Immunofluorescence double staining for F4/80 and TdT-mediated dUTP-biotin nick end labeling (TUNEL) and SOX10, β3-tubulin, and LC3

For immunofluorescence double staining, apoptosis was detected by the ApopTag Fluorescein In Situ Apoptosis Detection Kit (S7110; Chemicon International, Temecula, CA), as described previously^15^. And then, sections were incubated overnight with cell surface glycoprotein F4/80 (Serotec, Oxford, UK) and a Texas red-labeled secondary antibody and counterstained with 4,6-diamidino-2-phenylindole (DAPI). We also performed immunofluorescence double staining for SOX10 (Abcam, Cambridge, UK), β3-tubulin (1:50; Cell Signaling Technology, Danvers, MA), and LC3 (1:200; Sigma-Aldrich, St. Louis, MO, USA). The fluorescent images were examined under a laser scanning confocal microscope system (Carl Zeiss LSM 700, Oberkochen, Germany).

#### Electron microscopy

For transmission electron microscopy (TEM), sciatic nerves specimens were fixed in 4% paraformaldehyde and 2.5% glutaraldehyde in 0.1 M phosphate buffer overnight at 4 °C. After washing in 0.1 M phosphate buffer, the specimens were post-fixed with 1% osmium tetroxide in the same buffer for 1 h. The specimens were then dehydrated using a series of graded ethanol, exchanged through acetone, and embedded in Epon 812. Ultrathin sections (70–80 nm) were obtained by ultramicrotome (Leica Ultracut UCT, Leica, Germany) and were double stained with uranyl acetate and lead citrate and examined in transmission electron microscope (JEM 1010, Tokyo, Japan) at 60 kV. We measured areas of unmyelinated fiber, axonal diameters, *G* ratio, and the number of degenerative fiber using NIH Image J.

### Western blot analysis

The total proteins of the sciatic nerve tissues were extracted with a Pro-Prep Protein Extraction Solution (Intron Biotechnology, Gyeonggi-Do, Korea), following the manufacturer’s instructions. Western blot assay was performed with specific antibodies for CaSR (Thermo Fisher Scientific Inc, Waltham, MA, USA), CaMKKβ (Santa Cruz Biotechnology, Santa Cruz, CA, USA), total LKB1 (Cell Signaling Technology, Danvers, MA, USA**)**, phosphor-Ser^428^ LKB1 (Cell Signaling Technology, Danvers, MA, USA), total AMPK (Cell Signaling Technology, Danvers, MA, USA), phospho-Thr^172^ AMPK (1:2000; Cell Signaling Technology, Danvers, MA, USA), total eNOS (Cell Signaling Technology, Danvers, MA, USA), phospho-Ser^1177^ eNOS (Cell Signaling Technology, Danvers, MA, USA), PGC-1α (Novus Biologicals, Littleton, CO, USA), B cell leukemia/lymphoma 2 (Bcl-2) (Santa Cruz Biotechnology, Santa Cruz, CA, USA), BCL-2-associated X protein (Bax) (Santa Cruz Biotechnology, Santa Cruz, CA, USA), beclin-1 (Novus Biologicals, Littleton, CO), and LC-3 (Sigma-Aldrich, St. Louis, MO). After incubation with horseradish peroxidase-conjugated anti-mouse or anti-rabbit IgG (Cell Signaling Technology, Danvers, MA), target proteins were visualized by an enhanced chemiluminescence substrate (ECL Plus, GE Healthcare Bio-Sciences, Piscataway, NJ).

### HSC culture study

#### [Ca^++^]i measurement

HSCs were cultured in SC Medium (ScienCell Research Laboratories, San Diego, CA), as described previously^15^. Passages 4–8 were used in all experiments. The HSCs were exposed to low glucose (5 mmol/L d-glucose; low-glucose) or high glucose (40 mmol/L d-glucose), with or without the additional 24-h application of cinacalcet (15 and 100 nM). Calcium concentrations were determined based on the ratio of fura-2 fluorescence intensities at 340-nm excitation and 380-nm excitation. The ratio of 340/380 directly reflects the amount of [Ca^++^]i. The 340-nm fluorescence of fura-2 increases and the 380-nm of fura-2 decreases with increasing [Ca^++^]i. For [Ca^++^]i measurements, HSCs (20,000 cells/well) were plated on black 96-well plates with a clear bottom in complete medium. After 1 day, the cultures were serum-starved for 2 h in SC media. In the last 45 min of serum starvation, 5 mM FURA-2AM without Ca^++^ was added to the cells and then rinsed with Hank's Balanced Salt Solution (GibcoBRL, Grand Island, NY). FURA-2AM-loaded cells were sequentially excited at 340 and 380 nm by spectrophotometer microplate reader (Synergy MX; BioTek, Winooski, VT).

#### Immunofluorescence and western blot analyses in the HSCs

To evaluate the effects of cinacalcet on CsSR, CaMKKβ, phospho-Ser^428^ LKB1, and phospho-Thr^172^ AMPK expression, we performed immunofluorescence analysis with specific antibodies for CaSR, CaMKKβ, phospho-Ser^428^ LKB1, and phospho-Thr^172^ AMPK by using tyramide signal amplification fluorescence system and counterstained with DAPI. In addition, the total proteins of the HSCs were extracted with a Pro-Prep Protein Extraction Solution, following the manufacturer’s instructions. After incubation with horseradish peroxidase-conjugated anti-mouse or anti-rabbit IgG (Cell Signaling Technology, Danvers, MA), target proteins were visualized by an enhanced chemiluminescence substrate (ECL Plus, GE, Healthcare Bio-Science, Piscataway, NJ). To evaluate the anti-apoptotic effects of cinacalcet on HSCs in high-glucose medium, the number of TUNEL-positive cells was counted in 10 randomly chosen fields at a magnification of ×400. We also measured the concentration of NOx to quantify NO production in cell-culture media. The total NO_3_ and NO_2_ were quantified using the Nitric Oxide Assay Kit (Bio Vision, Mountain View, CA).

### HSCs with small interfering RNA (siRNA) transfection

siRNAs, targeted against CaMKKβ, LKB1, and scrambled siRNA (siRNA cont), were complexed with the transfection reagent (Lipofectamine 2000; Invitrogen, Carlsbad, CA), according to the manufacturer’s instructions. The sequences of the siRNAs are as follows: CaMKKβ, 5′-GGAUCUGAUCAAAGGCAUCTT-3′; LKB1, 5′-GGACUGACGUGUAGAACAATT-3′; and nonspecific scrambled siRNA, 5′-CCUACGCCACCAAUUUCGU-3′ (Bioneer, Daejeon, Korea). HSCs in 6-well plates were transfected with a final concentration of 50 nM CaMKKβ and LKB1 siRNAs for 24 h using the transfection reagent (Lipofectamine 2000) in Opti-MEM media (Gibco Life Technologies), according to the manufacturer’s instructions. After transfection, cells were treated with cinacalcet (5 nM) in high-glucose medium to evaluate the effects of siRNAs on HSC reactions.

### Data analysis

SPSS version 16 (SPSS. Inc., Chicago, IL) was used to conduct the statistical analysis. Group differences were evaluated using an analysis of variance with the Bonferroni correction. Non-normally distributed data were analyzed by Mann–Whitney *U* test. The results are expressed as mean ± SD. A *P* value of <0.05 was considered statistically significant.

## Results

### Body weight, blood HbA1c, glucose, iCa^++^, and PO_4_^3^^−^ levels

The body weights of *db/db* mice were greater than those of *db/m* mice in both the cinacalcet treatment and control groups at the end of the study (*p* < 0.001, Table 1). No changes in body weight were noted in *db/m* and *db/db* mice following the 12-week treatment with cinacalcet. HbA1c and fasting blood sugar concentrations were significantly higher in *db/db* mice than those in *db/m* mice in both the cinacalcet treatment and control groups (*p* < 0.001, Table 1). Interestingly, cinacalcet treatment did not change fasting blood glucose, HbA1c, or serum iCa^++^ and PO_4_^3−^ concentrations in *db/db* or *db/m* mice.

**Table 1:** Biochemical and physical characteristics of all the study groups

	*db/m* cont	*db/m*+cina	*db/db* cont	*db/db*+cina
Body wt (g)	31.9 ± 1.7	31.3 ± 2.0	55.7 ± 5.5^a^	54.5 ± 7.4^a^
HbA1c (%)	4.5 ± 0.3	4.5 ± 0.4	12.1 ± 1.3^a^	11.8 ± 1.4^a^
HbA1c (mmol/mol)	(26 ± 0.8)	(26 ± 0.9)	(109 ± 3.7^a^)	(105 ± 3.9^a^)
Glucose (mg/dl)	211 ± 25	202 ± 20	554 ± 39^a^	544 ± 54^a^
iCa^++^ (mmol/l)	1.29 ± 0.03	1.24 ± 0.05	1.33 ± 0.06	1.29 ± 0.07
PO_4_^3−^ (mmol/l)	3.8 ± 0.8	3.9 ± 0.9	4.0 ± 1.0	3.9 ± 0.8

*Ca* total calcium, *Cr* creatinine, *iCa*^*++*^ serum ionized Ca^++^

^a^*P* < 0.001 compared to the other groups (*n* = 8 in each experiment)

### Assessment of peripheral nerve function

#### Tactile response thresholds, motor nerve conduction latency, and action potential amplitude in the sciatic nerve

**Fig. 1:**
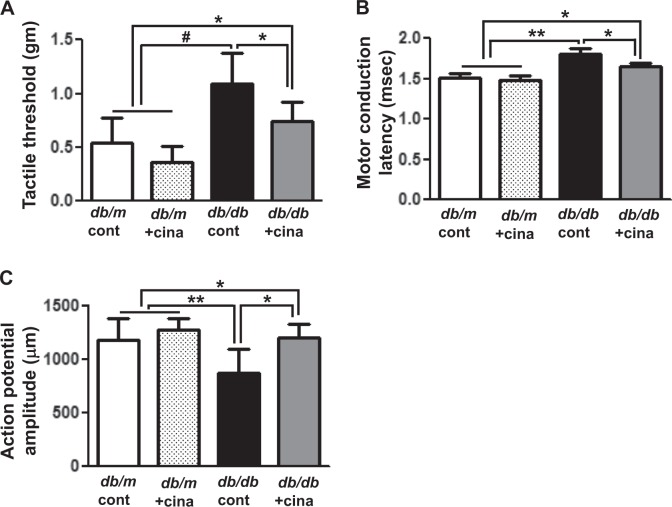
Cinacalcet improves sensory and motor functions of the sciatic nerve in *db/db* mice. **a**–**c** Effects of cinacalcet on the tactile threshold (**a**), motor conduction latency (**b**), and action potential amplitude (**c**) were determined at 20 weeks in *db/m* and *db/db* mice with or without cinacalcet treatment. (*n* = 8 in each groups) *p < 0.05, **p < 0.01, and ^#^*p* < 0.001 compared with the other groups

Tactile response thresholds (Fig. 1a, 1.09 ± 0.28 vs. 0.0.53 ± 0.23 g, *p* < 0.001) and MNCL (Fig. 1b, 1.81 ± 0.06 vs. 1.51 ± 0.05 ms, *p* < 0.01) were increased in *db/db* cont compared with those in *db/m* cont mice at the end of the 12-week study. Interestingly, cinacalcet treatment significantly improved the tactile response threshold and MNCL in *db/db* mice compared to those in *db/db* control (Fig. 1a, 0.74 ± 0.18 vs. 0.36 ± 0.14 g, *p* < 0.01 and Fig. 1b, 1.64 ± 0.05, vs. 1.48 ± 0.06 ms, *p* < 0.01, respectively) to the levels similar to those in *db/m* mice. Consistent with the improvement in the tactile response threshold and MNCL, cinacalcet treatment increased action potential amplitude in *db/db* mice (Fig. 1c, 873 ± 224 vs. 1199 ± 124 μm, *p* < 0.01). However, there were no changes in the tactile response, sciatic motor conduction latency, and action potential amplitude in the *db/m* mice treated with or without cinacalcet.

### Assessment of nerve pathology

#### Expressions of nerve fibrosis and Collagen IV (Col IV)

**Fig. 2:**
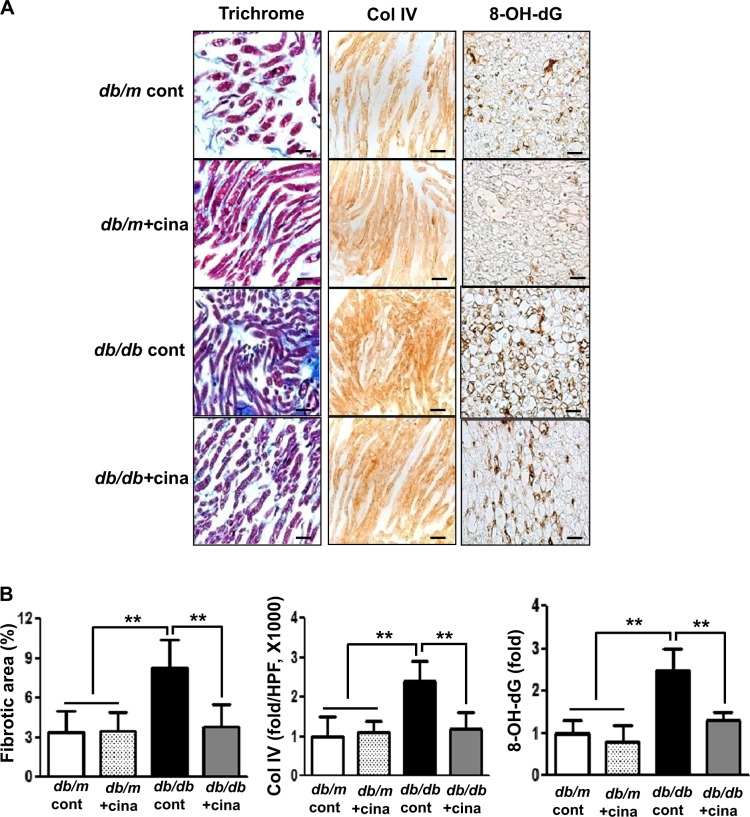

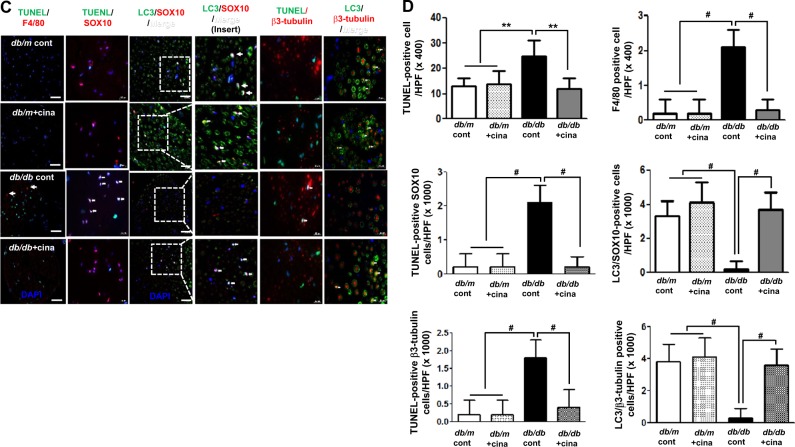
Cinacalcet attenuates fibrosis, inflammation, and apoptosis of the sciatic nerve in *db/db* mice. **a**–**d**. Nerve fibrosis, oxidative stress, inflammatory cell infiltration, and apoptosis in the sciatic nerves were determined at 20 weeks in *db/m* and *db/db* mice with or without cinacalcet treatment. Representative Masson’s trichrome staining and immunohistochemical staining for Col IV (**a**, **b**) and 8-hydroxy-deoxyguanosine (8-OH-dG), immunofluorescence for TUNEL, F4/80-positive cells, TUNEL-SOX10- and TUNEL-β3 tubulin-positive cells, and LC3-SOX10- and LC3-β3 tubulin-positive cells were determined (**b**). The white dotted line box indicates the area for each enlarged figure. The white arrows indicate TUNEL-SOX10- and TUNEL-β3 tubulin-positive cells and LC3-SOX10- and LC3-β3 tubulin-positive cells. The quantitative analyses of the results are shown (**d**, original magnification, ×1000). Scale bar = 10 μm (**a**, **c**). (*n* = 8 in each groups) **p < 0.01, and ^#^*p* < 0.001 compared with the other groups

The sciatic nerve in the *db/db* cont mice showed significant nerve fibrosis as reflected by increased trichrome-stained area when compared with that of *db/m* mice (Fig. 2a, b, 8.3 ± 2.1 vs. 3.4 ± 1.6%, *p* < 0.01) Immunohistochemical staining revealed increased expression of Col IV in the sciatic nerve of *db/db* cont compared to that of *db/m* cont mice (Fig. 2a, b, 2.4 ± 0.5 vs. 1.0 ± 0.5 folds, *p* < 0.01). Increased expression of Col IV and consistent increase in the extent of fibrotic area in the sciatic nerve of *db/db* control mice were improved to the levels similar to those of *db/m* mice with cinacalcet treatment (Fig. 2a, b, 3.8 ± 1.7% and 1.2 ± 0.4 folds, *p* < 0.01, respectively). Thus a 12-week cinacalcet treatment significantly improved fibrosis in the sciatic nerve of *db/db* mice.

#### 8-OH-dG, F4/80-positive cells, and TUNEL-positive cells in the sciatic nerve

The presence of 8-OH-dG-positive area was more prominent in *db/db* cont mice compared with that in *db/m* cont mice, reflecting increased amount of neuronal oxidative stress (Fig. 2a). However, cinacalcet treatment decreased the production of 8-OH-dG to the level comparable to that of *db/m* mice (Fig. 2b). We observed the number of TUNEL-positive neural cells, including SC and peripheral neuronal cells which are marked with SOX10 and β3-tubulin, respectively. Expression of TUNEL-positive cells was significantly increased in *db/db* cont compared with that in *db/m* cont mice (Fig. 2c, d, 25 ± 6 vs. 13 ± 3 cells/high-power field (HPF), *p* < 0.01). Cinacalcet treatment reduced the number of TUNEL-positive neural cells in *db/db* mice (12 ± 1.7 cells/HPF). Additionally, F4/80-positive inflammatory cell infiltration was severe in *db/db* mice when compared with that in non-diabetic *db/m* mice (2.1 ± 0.5 vs. 0.2 ± 0.4 cells/HPF, *p* < 0.01). Cinacalcet treatment reduced the number of F4/80-positive cells in *db/db* mice (0.2 ± 0.3 cells/HPF). There were no significant changes in the expression of TUNEL- and F4/80-positive cells in the sciatic nerve of non-diabetic mice with or without cinacalcet treatment. Significant decrease in the expression of LC3 in SOX10- and β3-tubulin-positive cells in diabetic control mice increased with cinacalcet treatment to the levels comparable to those of non-diabetic mice, reflecting the recovery of autophagy process (Fig. 2c, d).

#### Electron microscopy

**Fig. 3:**
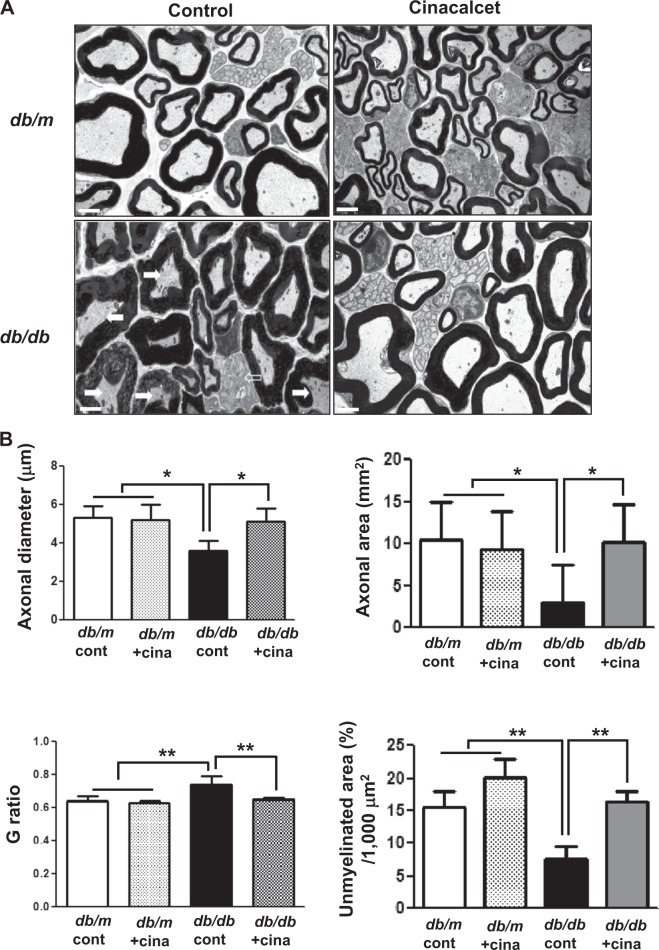
Cinacalcet increases the axonal area and area of unmyelinated fibers and decreases Schwann cell degeneration of the sciatic nerve in *db/db* mice. **a**, **b** The axonal area, area of unmyelinated fiber, and Schwann cell degeneration in the sciatic nerves were determined at 20 weeks in *db/m* and *db/db* mice. Representative electron microscopic images of the sciatic nerve bundles (×5000) (**a**). Marked decreases in the number of unmyelinated nerve bundles (open arrows) and prominent axonal degeneration (arrows) were observed in the *db/db* controls. These deficits were improved by the 12-week cinacalcet treatment in the *db/db*+cina mice (**a**). Quantitative analyses of axonal area and areas of unmyelinated fiber are shown (**b**). Scale bar = 2 μm (**a**). (*n* = 8 in each groups) *p < 0.05, **p < 0.01compared with the other groups

The ultrastructural features of sciatic nerves in *db/db* mice were characterized by severe myelin disruption with axonal shrinkage, degenerated SCs, and decreased amount of unmyelinated fibers with vacuolization when compared with those in *db/m* cont mice (Fig. 3a). Sciatic nerves from the *db/db* mice treated with cinacalcet displayed ultra-micro structures that resembled those from *db/m* control mice. Significant decreases in both axonal diameter and area and unmyelinated area of *db/db* mice were increased with cinacalcet treatment to the levels comparable to those of non-diabetic mice groups (*p* < 0.05 and *p* < 0.01, respectively; Fig. 3b). In contrast, increased *G* ratio in *db/db* mice was decreased with cinacalcet treatment, indicating optimized axonal myelination^24^ with improved functional and structural indices (*p* < 0.01; Fig. 3b).

### Expression levels of CaSR, CaMKKβ, phospho-Ser^428^ and total LKB1, phospho-Thr^172^ and total AMPK expression, PGC-1α, phospho-Ser^1177^ eNOS, Bcl-2, Bax, Beclin-1, and LC3-1 and -II in the sciatic nerve

**Fig. 4:**
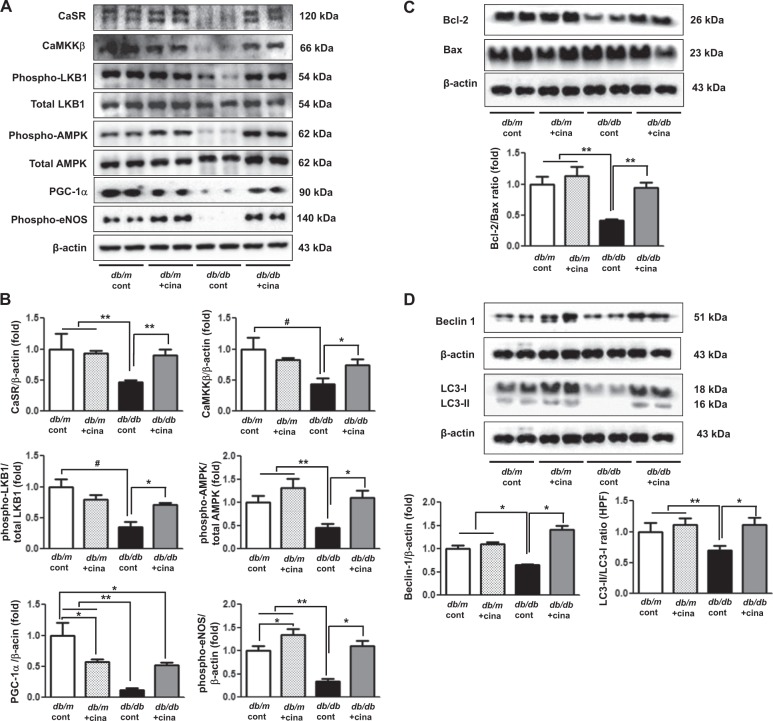
Cinacalcet upregulates CaSR, CaMKKβ, phospho-Ser^428^ LKB1, phospho-Thr^172^ AMPK, PGC-1α, phospho-Ser^1177^ eNOS, Bcl-1/Bax ratio, Beclin-1, and LC3-II/LC3-I ratio of the sciatic nerve in *db/db* mice. **a**, **b** The expression levels of CaSR, CaMKKβ, phospho-Ser^428^ LKB1, phospho-Thr^172^ AMPK, PGC-1α, phospho-Ser^1177^ eNOS, Bcl-1, Bax, Beclin-1, LC3-I, and LC3-II of the sciatic nerve were determined at 20 weeks in *db/m* and *db/db* mice. Cinacalcet treatment upregulates the expression of these intracellular signal pathway after the 12-week treatment in *db/db* mice. Representative western blot of CaSR, CaMKKβ, phospho-Ser^428^ LKB1, phospho-Thr^172^ AMPK, PGC-1α, phospho-Ser^1177^ eNOS, and β-actin (**a**) and quantitative analyses of the results are shown (**b**). **c**, **d** Representative western blot of Bcl-2 and Bax (**c**) and Beclin-1, LC3-I, and LC3-II (**d**) and their quantitative analyses of the results are shown (**c**, **d**, respectively). (*n* = 2 in each groups) *p < 0.05, **p < 0.01, and ^#^*p* < 0.001 compared with the other groups

On western blot analysis, CaSR, CaMKKβ, phospho-LKB1, phospho-AMPK, PGC-1α, and phospho-eNOS expression was significantly decreased in the sciatic nerve of *db/db* cont compared with that of *db/m* cont mice (Fig. 4a, b). These findings suggest that diabetes itself decreases the activation of CaSR-CaMKKβ and LKB1 phosphorylation, resulting in a decrease in AMPK phosphorylation, which seems to be related to the development of diabetic peripheral nerve damage. In contrast, cinacalcet treatment increased the expression of CaSR, CaMKKβ, phospho-Ser^428^ LKB1, and phospho-Thr^172^ AMPK, which subsequently recovered PGC-1α and eNOS expression in the sciatic nerve of *db/db* mice (Fig. 4a, b). For further evaluation of apoptosis and autophagy, we also measured the expression of Bcl-2/Bax ratio, Beclin-1, and LC3-II/LC3-I ratio in the sciatic nerves. Significant decreases in the expression of Bcl-2/Bax, Beclin-1, and LC3-II/LC3-I ratio in the sciatic nerve of *db/db* mice increased with cinacalcet treatment to the levels comparable to those of *db/m* mice (Fig. 4c, d).

### Effects of cinacalcet on [Ca^++^]i in HSCs

**Fig. 5:**
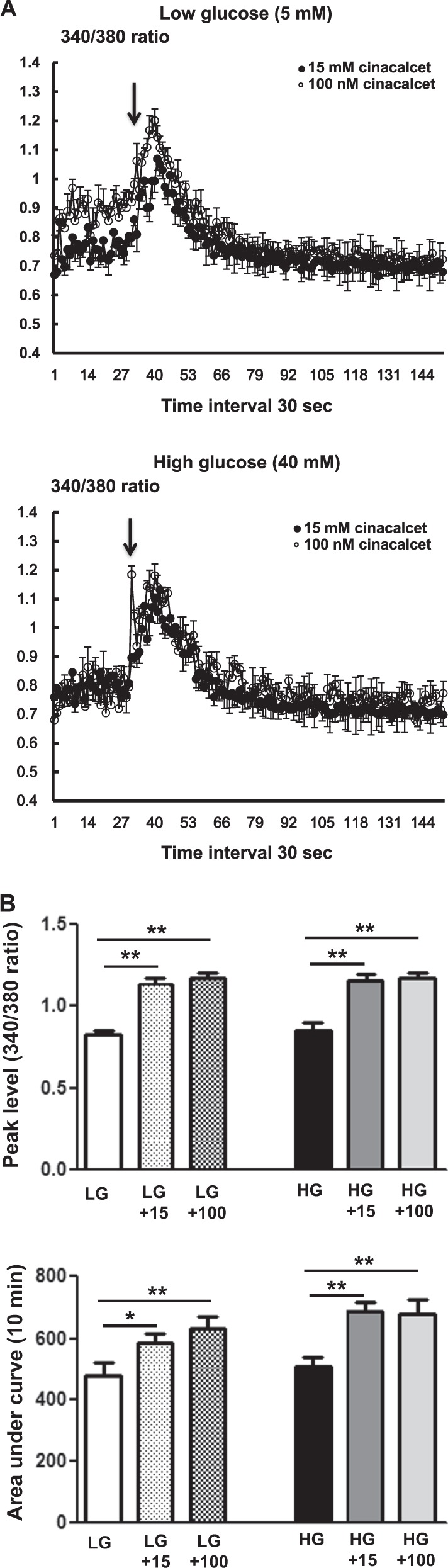
Cinacalcet increases [Ca^++^]i in HSCs grown in high-glucose medium. **a**, **b**. To determine whether the addition of cinacalcet might modulate [Ca^++^]i in HSCs, FURA-2AM-loaded human Schwann cells (HSCs) were stimulated using different concentrations (15, 100 nM) of cinacalcet in low-glucose (LG; 5 mmol/l d-glucose) or high-glucose (HG; 40 mmol/l d-glucose) medium. The area under curve (AUC) was estimated from the baseline of normalized data (at the point of injection) to a fluorescence level and between time points of injection (0 min) and 10 min. The peak of the curve was measured as the highest value of the curve. The peak amplitude and AUC of [Ca^++^]i were significantly increased by cinacalcet in dose-dependent manners (**a**, **b**) in both LG and HG media. In  **a**, the arrow denotes the administration of cinacalcet (15 and 100 nM, respectively). (*n* = 6 independent experiments in each experiments). *p < 0.05, **p < 0.01, and ^#^*p* < 0.001 compared with the other groups

**Fig. 6:**
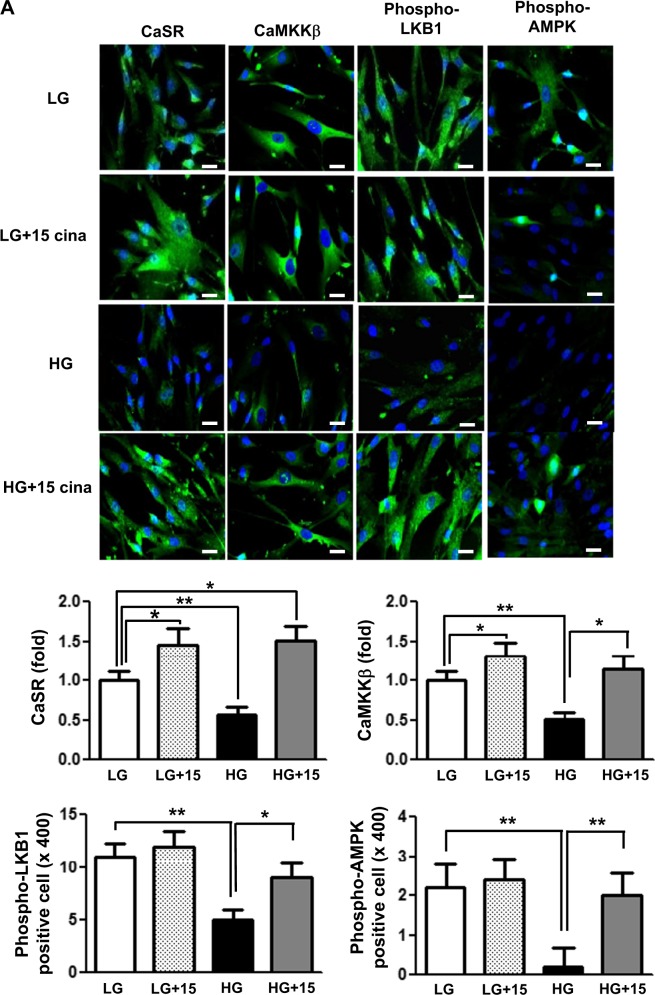

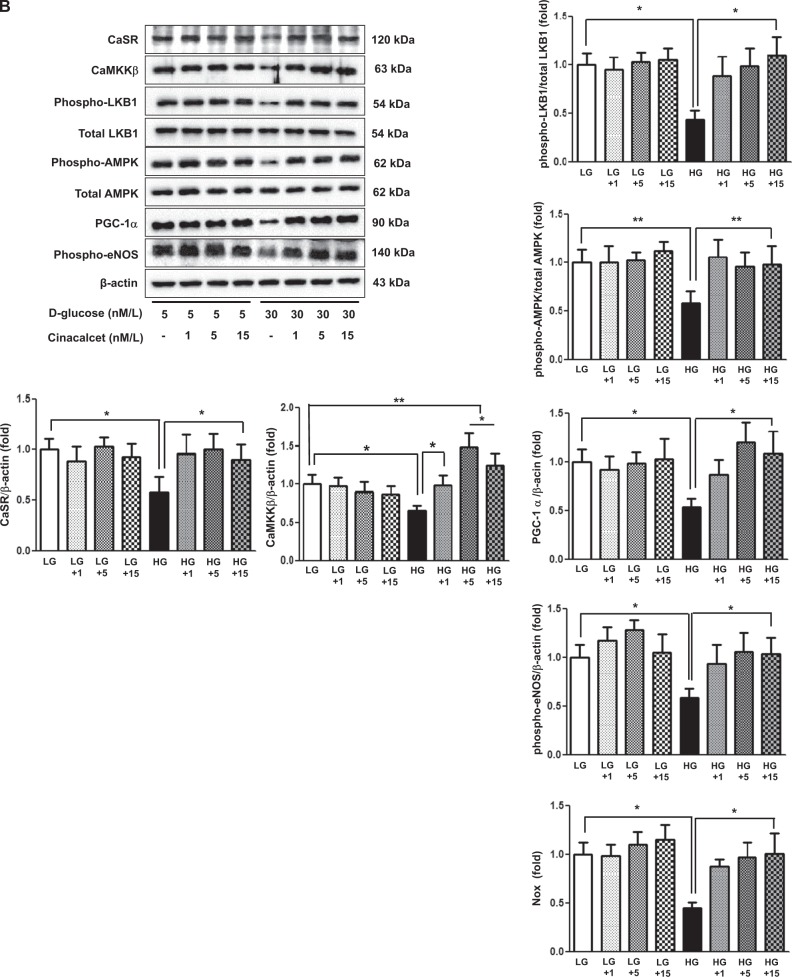

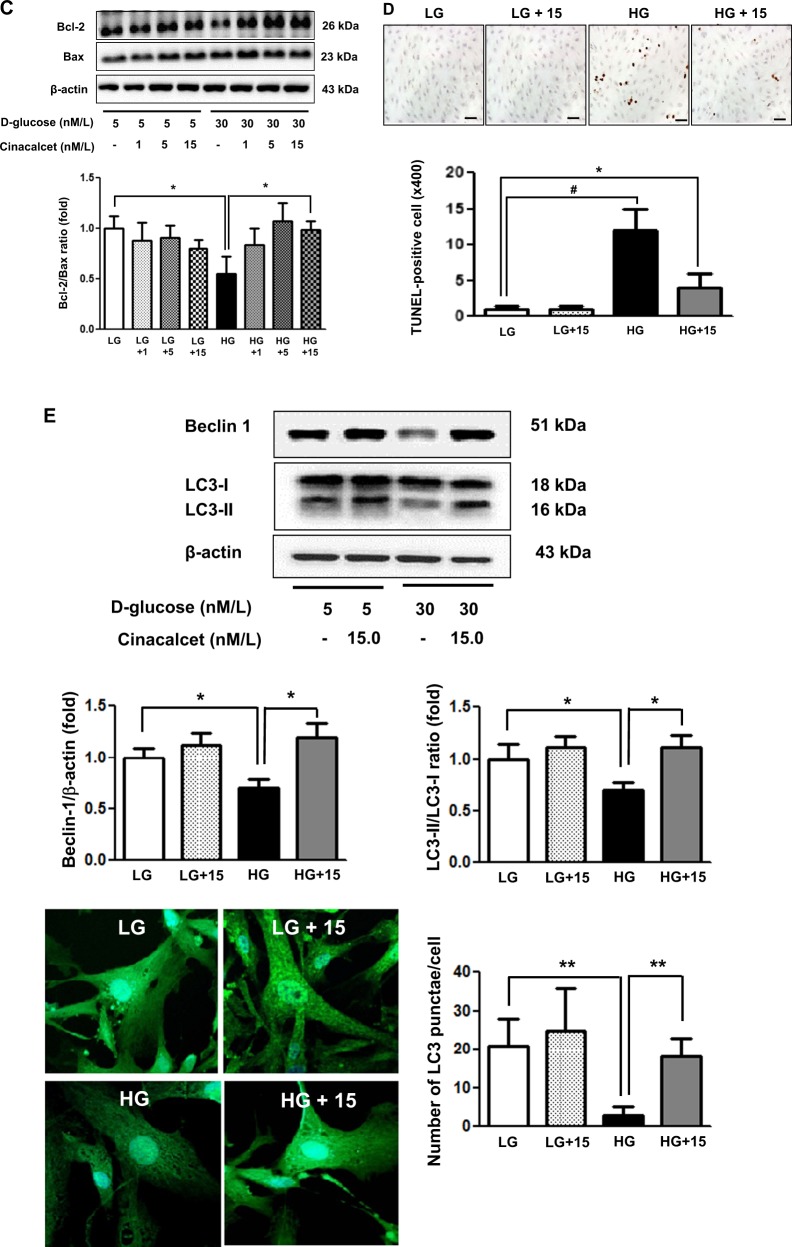
Cinacalcet activates intracellular CaSR, CaMKKβ, phospho-Ser^428^ LKB1, phospho-Thr^172^ AMPK, PGC-1α, phospho-Ser^1177^ eNOS, and NOx in HSCs grown in high-glucose medium, which prevents oxidative stress and apoptosis. **a** Immunofluorescence analysis was performed for CaSR, CaMKKβ, and phosphor-Ser^428^ LKB1 in the HSCs with or without cinacalcet treatment (15 nM; original magnification, ×400) and the quantitative analyses of the results are shown. Scale bars represent 30 μm. (*n* = 6 independent experiments in each experiments) (**a**). **b**, **c** The effect of cinacalcet on intracellular signals and apoptosis in the human Schwann cells (HSCs) cultured in low-glucose (LG; 5 mmol/l d-glucose) or high-glucose (HG; 40 mmol/l d-glucose) conditions without or with dose-dependent cinacalcet treatment (1, 5, 15 nM) were determined. CaSR, CaMKKα/β, total LKB1, phosphor-Ser^428^ LKB1, total AMPK, phospho-Thr^172^ AMPK, PGC-1α, phospho-Ser^1177^ eNOS, NOx, BCL-2, BAX, and β-actin levels were assessed in the cultured HSCs. Representative western blot analyses and quantitative analyses of CaSR, CaMKKα/β, total LKB1, phospho-Ser^428^ LKB1, total AMPK, phospho-Thr^172^ AMPK, PGC-1α, and phospho-Ser^1177^ eNOS are shown (**b**). The NOx concentrations from the supernatant of HSCs are shown (**b**). Representative western blot analyses and quantitative analyses of BCL-2 and BAX are shown (**c**). **d**, **e**. Marked increases in TUNEL-positive HSCs were observed in the HG medium compared to HG+15 group. The quantitative analyses of the results are shown (**d**). Representative western blot analyses and quantitative analyses of Beclin-1, LC3-1, and LC3-II are shown (**e**). The quantitative analyses of the results are shown (**e**). Marked decreases in the number of punctate in an SC were observed in the HG medium compared to HG+15 group (**e**). (*n* = 4 independent experiments in each experiments) *p < 0.05, **p < 0.01, and ^#^*p* < 0.001 compared with the other groups

It is well known that an increase in [Ca^++^]i is associated with the activation of CaMKK and LKB1, which are potent activators of AMPK. Therefore, we measured the effect of cinacalcet on [Ca^++^]i in the HSCs grown in either low- or high-glucose medium with or without cinacalcet. Interestingly, both 15 and 100 nM of cinacalcet significantly increased the peak [Ca^++^]i levels and its area under curve (Fig. 5a, b). We further evaluated the effects of cinacalcet on AMPK activation in cultured HSCs by immunofluorescence staining and western blot analysis. The intracellular LKB-1-AMPK-PGC-1α-eNOS signaling pathway is thought to play an important role in the maintenance of normal SC function. Thus we investigated the upstream signals of AMPK; significant decreases in CaSR, CaMKKβ, phospho-Ser^428^ LKB1, and phospho-Thr^172^ AMPK expression were noted in HSCs grown in high-glucose medium, resulting in subsequent decreases in PGC-1α and phospho-Ser^1177^eNOS expression, which are downstream targets of AMPK (Fig. 6a, b). Consistent with the intracellular signaling changes, decreased extracellular NOx concentration and Bcl-2/Bax ratio in high-glucose medium was increased significantly with cinacalcet treatment with subsequent decrease in TUNEL-positive cells in high-glucose medium (Fig. 6b−d). Such changes were not observed either in low-glucose medium or in osmotic control (not shown), suggesting that high-glucose medium takes part in the suppression of [Ca^++^]i-CaMKKβ-LKB-1-phospho-AMPK signaling and subsequent PGC-1α-phospho-eNOS-NOx axis in HSCs, which resulted in increased apoptotic cell deaths of HSCs. We also evaluated whether AMPK phosphorylation by cinacalcet preserves autophagy activity in SCs. The high-glucose-induced decreases in beclin-1, LC3-II/LC3-I ratio, and number of LC3 punctate in SCs were completely recovered by cinacalcet treatment to the levels similar to those of HSCs grown in low-glucose medium (Fig. 6e).

**Fig. 7:**
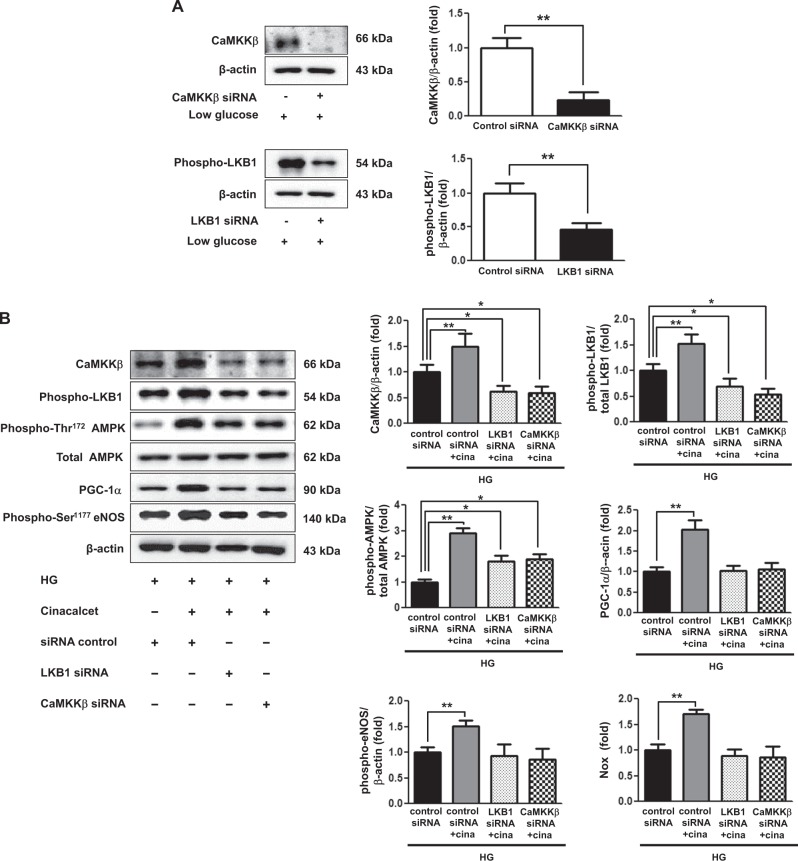
Cinacalcet activates intracellular CaMKKβ, phospho-Ser^428^ LKB1, phospho-Thr^172^ AMPK, PGC-1α and phospho-Ser^1177^ eNOS in HSCs grown in high-glucose medium. **a**, **b** The cultured HSCs were transfected with a final concentration of 50 nM *CaMKKβ* and *LKB1* siRNAs for 24 h by transfection reagent in low-glucose medium. Representative western blot analyses of CaMKKβ and phospho-Ser^428^ LKB1 and β-actin levels and the quantitative analyses of the results are also shown (**a**). **p < 0.01 compared with control siRNA. The cultured HSCs were transfected with a concentration of 50 nM *CaMKKβ* and *LKB1*siRNAs, respectively, for 24 h by transfection reagent and treated with cinacalcet (5 nM) in high-glucose medium, as well as CaMKKβ, phosphor-Ser^428^ LKB1, total AMPK, phospho-Thr^172^ AMPK, PGC-1α, phospho-Ser^1177^ eNOS, and β-actin levels and the quantitative analyses of the results are also shown (**b**). The NOx concentrations from the supernatant of HSCs are also shown (**b**). (*n* = 4 independent experiments in each experiments) *p < 0.05, **p < 0.01 compared with the other groups

Transfection of HSCs with CaMKKβ and LKB1 siRNAs suppressed the expression of CaMKKβ and phospho-Ser^428^ LKB1 by 80% and 60%, respectively, in low-glucose medium (Fig. 7a). Transfection with either *CaMKKβ* or *LKB1* siRNA resulted in the dual suppression in the expression of CaMKKβ and phospho-LKB1 despite cinacalcet treatment. Moreover, Cinacalcet treatment did not increase the expression of phospho-Thr^172^ AMPK-PGC-1α-phospho-Ser^1177^ eNOS-NOx signaling in HSCs when transfected with either CaMKKβ or LKB1 siRNA (Fig. 7b, c).

### Assessment of sciatic nerve function and phenotypes in 8-week-old *db/m* and *db/db* mice

**Fig. 8:**
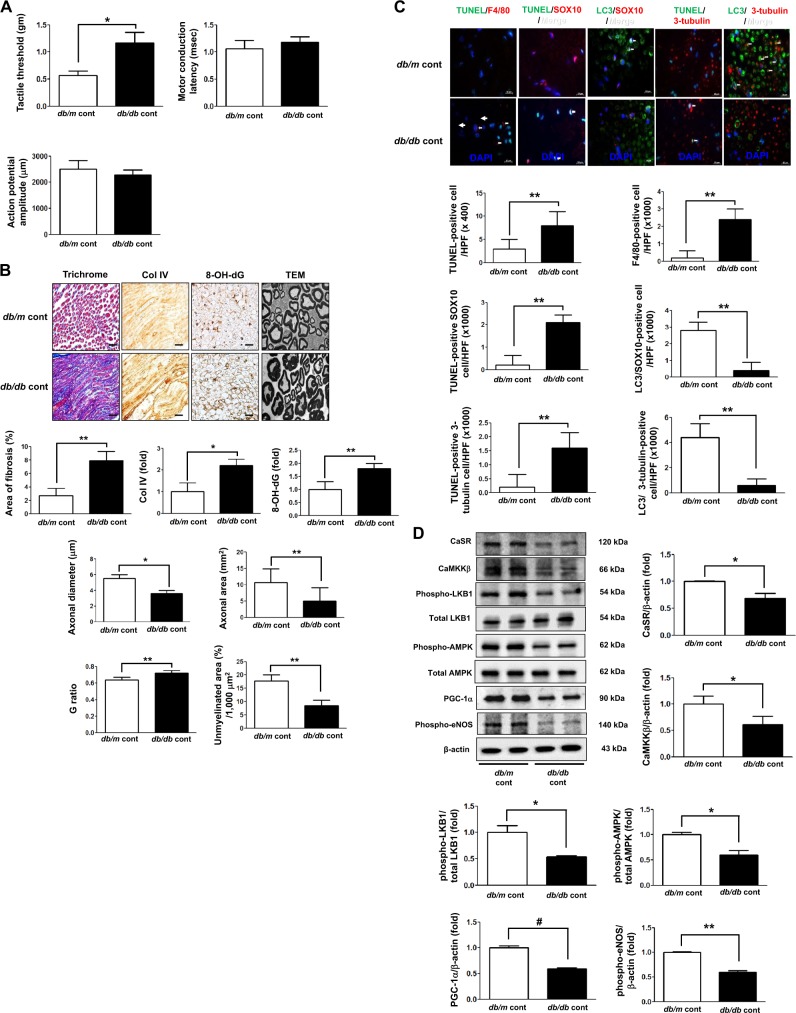
Functional and phenotypic changes in the sciatic nerve of 8-week-old *db/m* and *db/db* mice. **a** Effects of cinacalcet on the tactile threshold, motor conduction latency, and action potential amplitude were determined. **b** Nerve fibrosis (Masson’s trichrome and Col IV), oxidative stress (8-OH-dG), and the axonal diameter and area, the G ratio, and area of unmyelinated fiber in the sciatic nerves were determined. Representative electron microscopic images of the sciatic nerve bundles (×5000) are shown. Scale bars represent 2 μm. **c** Immunofluorescences for TUNEL, F4/80-positive cells, TUNEL-SOX10- and TUNEL-β3 tubulin-positive cells, and LC3-SOX10- and LC3-β3 tubulin-positive cells were determined. The white arrows indicate TUNEL-SOX10- and TUNEL-β3 tubulin-positive cells and LC3-SOX10- and LC3-β3-tubulin-positive cells, respectively. The quantitative analyses of the results are shown (**c**, original magnification, ×1000). Scale bars represent 10 μm (**b**, **c**). (*n* = 6 independent experiments in each experiments) *p < 0.05 and **p < 0.01 compared with the *db/m* cont group. **d** The expression levels of CaSR, CaMKKβ, phospho-Ser^428^ LKB1, phospho-Thr^172^ AMPK, PGC-1α, phospho-Ser^1177^ eNOS, Bcl-1, and β-actin of the sciatic nerve were determined. Representative western blot of CaSR, CaMKKβ, phospho-Ser^428^ LKB1, phospho-Thr^172^ AMPK, PGC-1α, phospho-Ser^1177^ eNOS, and β-actin and quantitative analyses of the results are shown (**d**). (*n* = 4 independent experiments in each experiments) *p < 0.05, **p < 0.01, and ^#^*p* < 0.001 compared with the other groups

To evaluate the effect of cinacalcet on the prevention and restoration of DPN, we investigated the functional and phenotypic changes in the sciatic nerves of 8-week-old *db/m* and *db/db* mice before the treatment. While tactile threshold was significantly increased in the sciatic nerve of *db/db* mice (*p* < 0.05), there were no significant differences in motor conduction latency and action potential amplitude between that of *db/m* and *db/db* mice (Fig. 8a). However, area of fibrosis, oxidative stress, and neuronal degeneration including decreased axonal diameter and area were prominent in the sciatic nerve of *db/db* mice with increased *G* ratio and decreased area for unmyelinated fibers (Fig. 8b). Furthermore, increased expression of F4/80- and TUNEL-positive cells and decreased expression of LC3-positive cells were noted in the sciatic nerve of *db/db* mice compared with those in *db/m* mice (Fig. 8c) and these changes were in line with decreased expression of CaSR-AMPK-eNOS signaling pathway in the same group (Fig. 8d).

## Discussion

The current study provides empirical evidences that cinacalcet improved sensorimotor function and restored damaged nerve phenotypes including nerve fibrosis and inflammation, axonal degeneration, loss of unmyelinated fibers, and apoptotic neuronal cell loss in the sciatic nerve of diabetic mice. Along with these changes, cinacalcet also restored defective autophagy activity in both SCs and peripheral nerve, which characterizes early-stage diabetic neuropathy. Activation of CaMKKβ and phosphorylation of LKB1 by cinacalcet increased phosphorylation of AMPK that subsequently activated PGC-1α and phospho-Ser^1177^ eNOS–NO and increased the ratio of Bcl-2/Bax, beclin-1, and LC3-II/LC3-I.

It is established that impaired [Ca^++^]i homeostasis is implicated in the development of DPN^25,26^. Previous studies supported the notion that deranged Ca^++^homeostasis is attributable to impaired sarco/endoplasmic reticulum calcium ATPase pumps located in the endoplasmic reticulum membrane^19^. Therapy with low-dose insulin and neurotrophin-3 restored resting Ca^++^ levels from intracellular stores, signifying that altered calcium homeostasis could be an early molecular marker linked to the onset of diabetic sensory neuropathy^27,28^. In the current study, cinacalcet treatment increased the expression of CaSR and [Ca^++^]i in cytoplasm in association with subsequent increase in the expression of CaMKKβ and LKB-1 in the diabetic animals and cultured SCs that was independent of adenylate energy balance, such as AMP/ATP and ADP/ATP ratios. More importantly, transfection with either *CaMKKβ* or *LKB1* siRNA resulted in the dual suppression in the expression of both CaMKKβ and LKB1 and their downstream phospho-Thr^172^ AMPK, PGC-1α, and phosphor-Ser^1177^ eNOS signaling in HSCs cultured in high-glucose medium. The results signify the dual activation of CaMKKβ and LKB1 as a prerequisite and that the interaction between the two upstream kinases is required for further enhancement of their downstream effectors by cinacalcet treatment.

AMPK is a major downstream effector of its upstream kinases that plays a key role in cell survival and death in response to metabolic stress. The role of AMPK activation in restoring nerve function, preventing, and even reversing pathological pain associated with DPN is implicated in various studies; we previously demonstrated that fenofibrate treatment ameliorated neuronal damage in the sciatic nerve of type 2 diabetic mice by activating the PPARα-AMPK-PGC-1α-eNOS pathway^15^. Moreover, mitochondrial dysfunction in DPN is characterized by maladaptation in such metabolic signaling pathway as AMPK/sirtuin–PGC-1α axis that contributes to the diminishment of axonal regeneration capacity^29^. In line with this, diabetes-associated alterations in the peripheral nerve phenotype and concomitant development of sensorimotor dysfunction were accompanied by decreased expression of AMPK-PGC-1α-eNOS signaling and this was ameliorated by cinacalcet treatment through the upregulation of AMPK-eNOS phosphorylation in the sciatic nerve of diabetic mice.

One favorable and potential downstream mediator of AMPK signaling is NO^30^. Neuronal damages may be associated with secondary deficits in endothelial function resulting from impaired NO synthesis, release of NO upon endothelial injury by oxidative stress, and increased free radical activity. Cinacalcet’s favorable effects on preventing sensorimotor dysfunction and neuronal damage may be implemented by AMPK-induced modulation of eNOS and enhancement of NOx production, which preserves peripheral nerve, especially SCs, and promotes endothelial cell survival and function.

To explore the cellular fate of peripheral nerves associated with enhanced eNOS–NOx activation, we determined the degree of autophagic activity as represented by LC3-II/LC3-I. LC3-II serves as a molecular biomarker for the assessment of autophagic activity. Impaired autophagic activity participates in the development of a variety of disease including neurodegenerative disorders probably due to the accumulation of damaged molecules and organelles that promotes cell death. On the other hand, excessive autophagy could also result in cell death and dysfunction by facilitating apoptosis with potential clinical significance, and therefore, the proportional contribution of autophagy^31^ and apoptosis and the balance between the two counteracting functions is essential in promoting cell viability and further maintaining the functional and phenotypic integrity of peripheral nerves in diabetes. In this aspect, cinacalcet may exhibit its potential as a means to promote cell survival by enhancing autophagy and attenuating apoptosis.

**Fig. 9:**
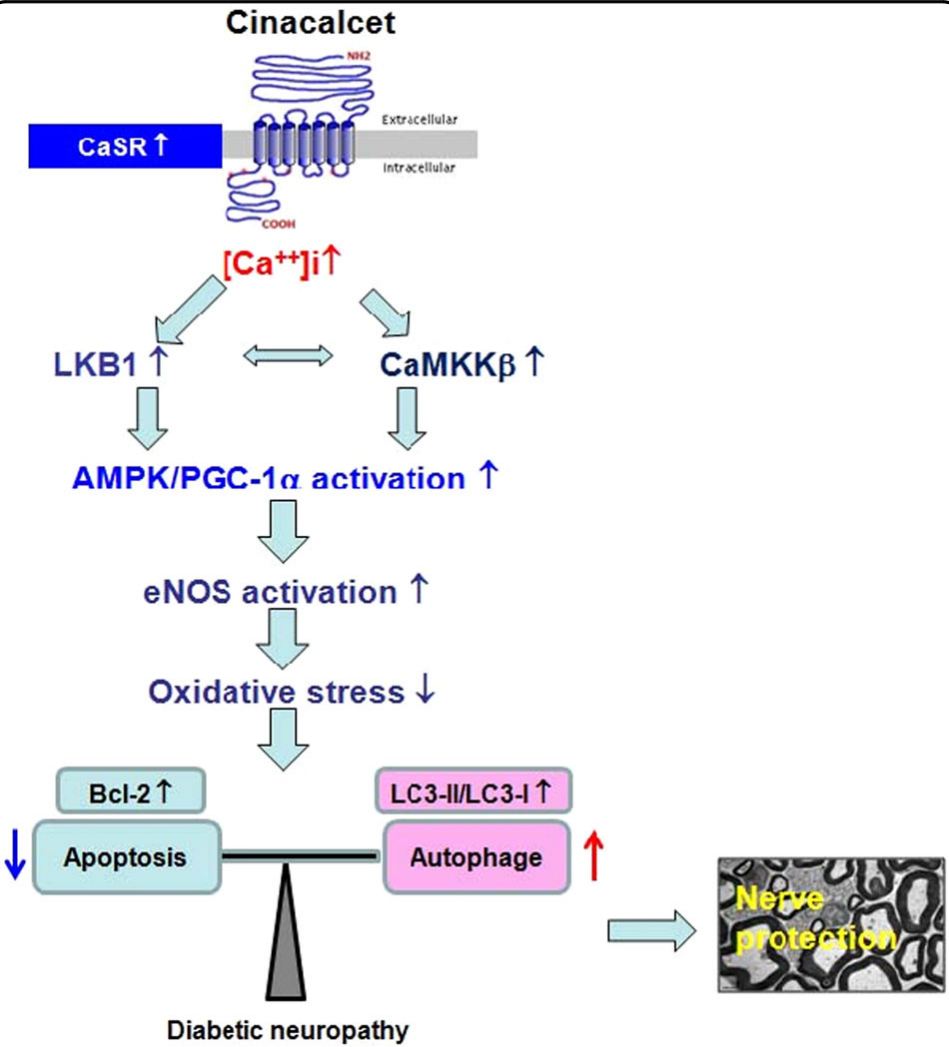
The proposed role of cinacalcet in diabetic peripheral neuropathy and the interplay between cinacalcet and peripheral nerve injury in type 2 diabetes. AMPK AMP-activated protein kinase, CaMKKβ Ca^2+^/calmodulin-dependent protein kinase kinase-β, eNOS endothelial nitric oxide synthase, LKB1 liver kinase B1, [Ca^++^]i intracellular Ca^++^

In summary, this study strongly suggests a favorable effect of cinacalcet in DPN by enhancing the [Ca^++^]i-CaMKKβ-LKB1 pathway and its downstream effectors AMPK-PGC-1α-eNOS, which may prevent the sciatic nerve injury from diabetes-induced oxidative stress through not only decreased apoptosis but also increased autophagy activity (Fig. 9). Therefore, cinacalcet-induced AMPK activation underscores the modulation of autophagy and apoptosis in the sciatic nerve and may be a promising therapeutic means to deter and prevent the progression of DPN.
